# Bayesian model averaging for predicting factors associated with length of COVID-19 hospitalization

**DOI:** 10.1186/s12874-023-01981-x

**Published:** 2023-07-06

**Authors:** Shabnam Bahrami, Karimollah Hajian-Tilaki, Masomeh Bayani, Mohammad Chehrazi, Zahra Mohamadi-Pirouz, Abazar Amoozadeh

**Affiliations:** 1 Student Research Center, Research Institute, Babol University of Medical Sciences, Babol, Iran; 2 Department of Biostatistics and Epidemiology, School of Public Health, Babol University of Medical Sciences, Babol, Iran; 3 Social Determinants of Health Research Center, Research Institute, Babol University of Medical Sciences, Babol, Iran; 4 Department of Infectious Diseases, Ayatollah Rohani Hospital, Babol University of Medical Sciences, Babol, Iran; 5 Neonatal Research Unit, Imperial College London, Exhibition Rd, South Kensington, London, SW7 2BX UK

**Keywords:** AIC, GBDT, Bayesian model averaging, BIC, COVID-19, Length of hospital stay, Markov Chain Monte Carlo (MCMC), Occam's Window, Stepwise

## Abstract

**Introduction:**

The length of hospital stay (LOHS) caused by COVID-19 has imposed a financial burden, and cost on the healthcare service system and a high psychological burden on patients and health workers. The purpose of this study is to adopt the Bayesian model averaging (BMA) based on linear regression models and to determine the predictors of the LOHS of COVID-19.

**Methods:**

In this historical cohort study, from 5100 COVID-19 patients who had registered in the hospital database, 4996 patients were eligible to enter the study. The data included demographic, clinical, biomarkers, and LOHS. Factors affecting the LOHS were fitted in six models, including the stepwise method, AIC, BIC in classical linear regression models, two BMA using Occam's Window and Markov Chain Monte Carlo (MCMC) methods, and GBDT algorithm, a new method of machine learning.

**Results:**

The average length of hospitalization was 6.7 ± 5.7 days. In fitting classical linear models, both stepwise and AIC methods (*R* ^2^ = 0.168 and adjusted *R* ^2^ = 0.165) performed better than BIC (*R* ^2^ = 0.160 and adjusted = 0.158). In fitting the BMA, Occam's Window model has performed better than MCMC with *R* ^2^ = 0.174. The GBDT method with the value of *R* ^2^ = 0.64, has performed worse than the BMA in the testing dataset but not in the training dataset. Based on the six fitted models, hospitalized in ICU, respiratory distress, age, diabetes, CRP, PO2, WBC, AST, BUN, and NLR were associated significantly with predicting LOHS of COVID-19.

**Conclusion:**

The BMA with Occam's Window method has a better fit and better performance in predicting affecting factors on the LOHS in the testing dataset than other models.

## Introduction

In December 2019, a cluster of pneumonia that was later shown to be a type of acute respiratory disease of a novel coronavirus (SARS-CoV-2), subsequently known worldwide as COVID-19, appeared in China [1]. The severity of the disease of covid-19 is very different between patients. In some of these patients, they may recover by receiving outpatient medical care and prescribing medicine at home. But, some others may be hospitalized and receive emergency care. And even some affected people do not respond to treatment and die [1, 2]. The COVID-19 pandemic has forced researchers around the world to find possible strategies to stop its transmission and factors affecting the disease. About 10% of patients may need to be hospitalized for emergency care and changing of their clinical conditions and thus the LOHS may not be predicted by physical conclusively.

There is still a gap of knowledge to predict the clinical changes in the severity of disease and the length of hospital stay (LOHS) [3]. The LOHS is the number of days a patient stays in the hospital. LOHS has long been used as a benchmark for hospitals to improve patient care. LOHS for COVID-19 may vary between 2 and 50 days between patients [4]. The length of hospitalization was different in different studies. For example, in the study of Birhanu et al. (2022) in Ethiopia, its average was 12 days [5] and in another study by Maj et al. (2021) in India it was reported to 9 days and increased with aging [6]. In a systematic review conducted by Tian et al. (2020), diabetes was one of the underlying diseases that had an important impact on the length of hospitalization [7].

In order to understand the factors associated with predicting the LOHS, different methods of statistical analysis have been used in the past few decades [2]. Among these methods, we can mention machine-learning methods. Models such as the decision tree (DT) can specify the relative importance of different explanatory variables related to the response variables, but the structure of the decision tree depends to a large extent on the data, which may lead to instability in the estimates [8]. The gradient-boosting decision tree (GBDT) model has superior performance in model interpretation and prediction accuracy compared to conventional DT models. In this model, the possible error is minimized by repeated modeling algorithm, in this sense, the estimation may be superior to other ML algorithms and the uncerainity of model is considered to some extend [9]. Classical linear regression and generalized linear models, as traditional methods, are used for predicting dependent variables given a set of explanatory variables. However, these long-established approaches generally do not consider the uncertainty of the model.

A Bayesian method to deal with the problem of model uncertainty is the Bayesian model averaging (BMA) [10]. BMA proposed by Draper provided a statistical theory basis to solve the problem of model uncertainty in econometric modeling [11]. This approach was implemented in R software programs, BMA package. This package is quite general and allows the Bayesian model to perform averaging of linear models, and generalized linear models with flexible management of initial parameters have it. In this study, we adopted the BMA approach as a tool to optimize the predictive performance of common statistical models used in large-scale data to achieve higher certainty in examining factors predicting the LOHS of patients with COVID-19. While most of the available information was analyzed through traditional regression models which are more exposed to uncertainty, and so far as there are no data to compare BMA methods with a new method of GBDT algorithm in predicting the length of hospitalization of COVID-19. Thus, this study aims to use the BMA method to estimate parameters with higher certainty in modeling the length of hospitalization of COVID-19 and to compare with traditional regression methods and GBDT algorithm as well.

In BMA method, Occam's Window algorithm can be used to obtain a small set of models that can be used to calculate the average model based on them. The second method is a Markov Chain Monte Carlo approach that directly approximates the exact integral solution of the equations related to the posterior distribution of the parameter of interest, which mostly does not have a closed form [12, 13].

## Methodology

### Study design

In this historical cohort study, the population consisted of patients who were diagnosed with COVID-19 and were admitted to Rouhani hospital in Babol, the north of Iran, during 2020-2021. The diagnosis was confirmed with clinical and para-clinical pieces of evidence by an infectious specialist.

### Participants

**Fig. 1:**
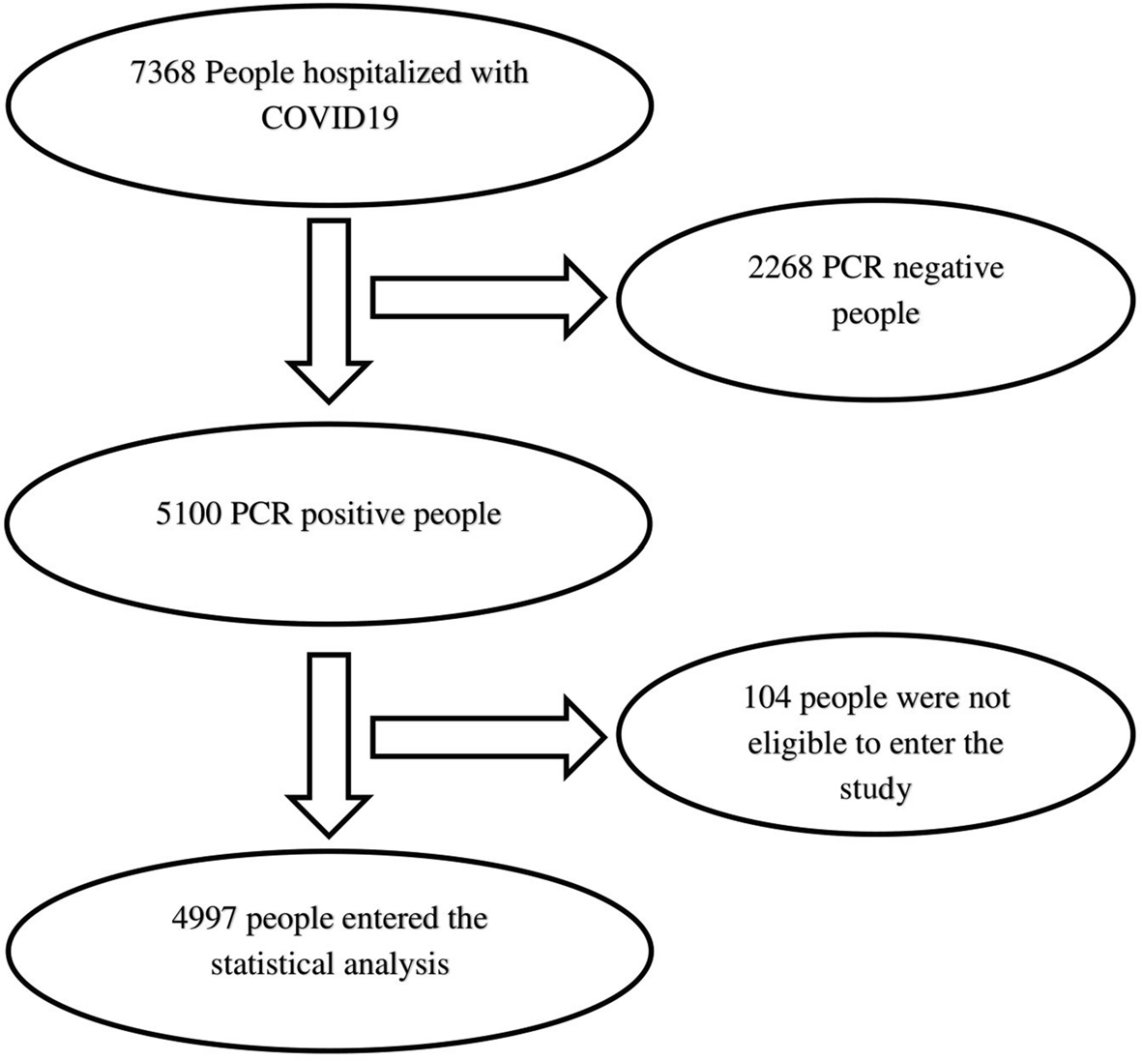
The flow chart of selecting of participants in the final sample

The investigated sample included 5100 people with Covid-19 affliction who had a positive PCR test result. Their demographic, clinical, and para-clinical information, and discharge status have been recorded in the HIS database of Rouhani Hospital and the MCMC database of Babol Health Center. In inclusion criteria, men and women over 18 with confirmed COVID-19 were eligible for the study. All hospitalized patients included in the study, a first-episode new crown. Individuals whose file information was incomplete, their required data was not recorded, cases of disagreement of the national patient code in the linkage of the MCMC database of the health center and the HIS database of Ayatollah Rouhani Hospital, and those who were hospitalized in the emergency room for less than 24 hours were excluded from the study. Out of 5100 CPR-positive participants, 104 cases were not eligible to enter the study. Thus, 4996 people were included in the statistical analysis. The flow chart of selection of participants was shown in Fig. 1.

### Data collection procedure

In this study, the linkage of two sets of databases of registered patients hospitalized with COVID-19 was used. The two sets were linked with the R software program, using the national code of hospitalizedpatients. The data includes biological markers such as WBC, ALT, AST, ALP, NLR, ESR, CRP, BUN, and PO2, some background diseases such as diabetes, asthma, COPD, chronic nervous disorders, immunodeficiency, HTN, and other chronic disorders. The detection time of all biomarkers was the first day of admission as baseline characteristics. In addition, the clinical symptoms in the first day of hospitalization such as fever, cough, muscle pain, respiratory distress, level of consciousness, smell, taste, seizure, abdominal pain, nausea, vomiting, anorexia, headache, dizziness, chest pain, dermatitis, and demographics data such as age and gender, as well as hospitalization at ICU, the duration of LOHS, and the discharge status from the hospital were extracted from the database.

### Ethical considerations

All the patients’ data were extracted from the database. The study protocol was approved by the ethics committee of Babol university of medical sciences with the ethics ID code: IR.MUBABOL.HRI.REC.1401.148**.**

### Multiple imputation of missing data

Multiple imputations of missing data were performed using R software from the Mice package. When missing data is due to a random mechanism, multiple imputations can be applied with different approaches. The fully conditional specification (FCS) and joint modeling (JM) methods are the most common approaches. In the multiple imputations of the JM model, the missing values of all variables are calculated simultaneously using a statistical model of joint probability functions. The FCS method, unlike JM, uses the joint distribution of the variables, but it estimates with a set of multiple imputation univariate conditional models. Considering that the JM method uses only one multivariable model, it is easier to use. In contrast, the FCS method is more flexible when there are a large number of variables with missing data because it considers a separate conditional model for each variable. The FCS method is more convenient and realistic to JM and it provides a highly flexible and behaves very well in statistical properties and less bias than complete-case analysis [14]. In the present study, the FCS method was used for missing data imputation.

### Statistical analysis

We used SPSS 26, STATA 15, and R software in the implementation of statistical models. In the first step, descriptive statistics and frequency distribution were performed on the entire data. In bivariate analysis, the LOHS was categorized as ≤ 5 days and > 5 days. According to the treatment protocol of Ministry of Health and Medical Education of Iran, the length of hospitalization with the first line main drugs was 5 days in management of COVID-19, and in some cases, the length of hospitalization lasted more than 5 days. The relationship between the quantitative variables of biomarkers and the dichotomized length of hospitalization, two independent samples t-tests, and the relationship between the underlying disease with the dichotomized length of hospitalization was determined using the Chi-square test. Then, the data was divided into two parts: training (80%) and testing (20%). Next, to examine the relationship between the quantitative response variable of the length of hospitalization with the independent variables in the study, the classical linear regression models such as stepwise, AIC, BIC, and Bayesian models averaging such as Occam's Window and Markov chain Monte Carlo (MCMC) and also the machine learning model like GBDT were fitted to 80% of the training data. Although the relation between blood test values and LOHS is dynamic and the level of biomarkers may change over the hospitalization, for avoiding the complexity of models, we built the regression models using clinical data and biomarkers that were measured on the first day of hospitalization. The fitted models were assessed by *R*^2^ and adjusted *R*^2^ index in training data. Then, the performance of the models was evaluated using both 80% of the training dataset and 20% of the testing dataset. The calibration chart, the average percentage of errors of different models, the average errors, and also the $MSE=\frac{\sum {\left(y-\widehat{y}\right)}^{2}}{N}$ were calculated for each model. Finally, the results were compared in different models.

#### Overview of linear regression models in a stepwise method

In the current study, linear regression analysis was used to investigate the effect of several independent variables on the LOHS as a dependent variable. In fact, in stepwise regression, all independent variables are included in the model, and those that do not have much effect on the dependent variable are removed from the model in a stepwise fashion. The regression method is performed stepwise from the backward method and forward method. In the forward method, first, there is no variable in the model and the first variable that enters the model has the highest correlation with the dependent variable. If after running the regression model, the significance value of the statistic is acceptable, the variable remains in the model. Next, the second variable that has the highest partial correlation with the dependent variable is entered into the model and the regression model is executed. This process continues until the significant value of the variables in the model does not exceed the desired level. In the backward method, first, all the variables are entered into the model; then in a stepwise fashion, the variable that is not at an acceptable level of significance is removed from the model. In this method, the execution continues until the last variable with the lowest amount of statistics is removed from the model [15]. The form of the linear regression model given a set of explanatory variables is as follows:

$$\widehat{y}={\alpha +\beta }_{1}{X}_{(age)}+{\beta }_{2}{X}_{(ALP)}+{\beta }_{3}{X}_{(ALT)}+\cdots$$

#### AIC, BIC criteria in the linear regression model

One of the most important criteria for choosing a better model is AIC (Akaike information criterion). This criterion can be used to compare models. The AIC formula estimated by Sakamoto in 1999 [16] is as follows:

$$AIC=-2(\log\;likelihood)+2p$$

Where p is the number of model parameters in the comparison of models, the model with a lower AIC value is selected as the best model [15].

BIC (Bayesian information criterion) criterion suggested by Schwartz in 1978 [17] is similar to AIC. In which, in addition to the number of parameters p, it also includes the sample size n.

$$BIC=-2\left(\log\;likelihood\right)+\ln(n)p$$

Similar to AIC, in the comparison of models, a model with a lower BIC value is selected as a better model. The form of the regression equation based on BIC and AIC criteria is similar as stepwise method.

#### Overview of Bayesian model averaging

If $M=\left\{{M}_{1}. {M}_{2}. \dots . {M}_{K}\right\}$ represents the set of all imputed models under consideration, and if ∆ is the parameter of interest, in future prediction the posterior probability should be estimated over a period of time. Then, the posterior probability distribution of ∆ parameter for the data D is as follows:

1 $$\mathrm{Pr}\left(\Delta |D\right)=\sum_{k=1}^{K}\mathrm{Pr}\left(\Delta |{M}_{k}.D\right)\mathrm{Pr}\left({M}_{k}|D\right).$$

$\mathrm{Pr}\left(\Delta |D\right)$ is the Bayesian averaging of the posterior probability ∆ under model with weighting based on the posterior probability distribution. In the above equation, $\mathrm{Pr}\left({M}_{k}|D\right)$ is the posterior distribution of the ${M}_{k}$ model:

2 $$\mathrm{Pr}\left({M}_{k}|D\right)=\frac{\mathrm{Pr}\left(D|{M}_{k}\right)\mathrm{Pr}({M}_{k})}{\sum_{l=1}^{K}\mathrm{Pr}\left(D|{M}_{l}\right)\mathrm{Pr}({M}_{l})}.$$

where

3 $$\mathrm{Pr}\left(D|{M}_{k}\right)=\int \mathrm{Pr}\left(D|{\theta }_{k}.{M}_{k}\right)\mathrm{Pr}\left({\theta }_{k}|{M}_{k}\right)d{\theta }_{k }.$$

In the above equations, $\mathrm{Pr}\left({M}_{k}|D\right)$ is the marginal probability of model${M}_{k}$, $\mathrm{Pr}\left({\theta }_{k}|{M}_{k}\right)$ in the prior distribution, ${\theta }_{k}$ is the vector of ${M}_{k}$ parameters and $\mathrm{Pr}({M}_{k})$ is the prior probability of K model. the Bayesian averaging of this method, which is performed on all models, can provide better forecasting ability. Because

4 $$-E\left[\mathrm{log}\left\{\sum_{k=1}^{K}\mathrm{Pr}\left(\Delta |{M}_{k}.D\right)\mathrm{Pr}\left({M}_{k}|D\right)\right\}\right]\le -E\left[\mathrm{log}\left\{\mathrm{Pr}\left(\Delta |{M}_{j}.D\right)\right\}\right] \left(j=1.\dots .K\right).$$

The above inequality shows that the BMA is better than the univariate model. According to the following equation:

5 $${D}_{KL}(P|\left|Q\right)=\sum_{x\epsilon X}P\left(x\right)\mathrm{log}\left(\frac{P\left(x\right)}{Q\left(x\right)}\right).$$

In this equation P(x), Q(x) can have a normal distribution or not. To compare distributions, we can compare their relative entropy (amount of information). If their entropy is close to zero (${D}_{KL}$ tends to zero), it means that their information P(x), Q(x) is similar. Otherwise, it would have a distance from zero. Therefore, the BMA entropy is better than the single model [18]. In the present study, in the implementation of the BMA, the two methods of Occam's Window and Markov Chain Monte Carlo (MCMC) were used.

#### Occam's Window method

Now, if there are p independent predictor parameters in a problem, the number of models (k) is equal to $k={2}^{p}$ (in the absence of other restrictions). But it is likely that some of these models are supported by very little data. Therefore, it is better to perform Bayesian averaging on the best models. Instead of applying the Bayesian averaging over all ${2}^{p}$ possible models. Occam's Window is a two-step method to find the best subset of predictors in a linear regression model. In this method, we limit the large set of predictors to a small number of predictors to provide a more accurate prediction.

In the first step, most of the models in equation (1) have been discredited because they predict the data much less than the best models, so they should be discarded and should not be included in equation (1). Therefore, the selected model for Bayesian averaging to set A' in equation (6) is limited.

6 $${A}^{^{\prime}}=\left\{{M}_{k}:\frac{{max}_{l}\left\{\mathrm{Pr}({M}_{l}|D\right\}}{\mathrm{Pr}({M}_{k}|D)}\le C\right\}.$$

Therefore, ${M}_{k}$ are the models that apply in the above conditions. C is a constant measure whose value is chosen depending on the subject. The number of models in this method increases with decreasing the value of C. In the current analysis, the value of C was 20 by default. In the second step, it removes the models that have less support from the data. According to the following related constraint, many models are removed.

7 $$\begin{array}{c}B=\left\{{M}_{k}:\exists {M}_{L}\in M.{M}_{L}\subset {M}_{K}.\frac{\mathrm{Pr}({M}_{L}|D)}{\mathrm{Pr}({M}_{K}|D)}>1\right\} \\ A={A}^{^{\prime}}/B\in M.\end{array}$$

The models are replaced in equation (1). Therefore:

$$\mathrm{Pr}\left(\Delta |D\right)=\frac{{\sum }_{{M}_{k}\in A}\mathrm{Pr}\left(\Delta \right|{M}_{k}.D) \mathrm{Pr}(D\left|{M}_{k}\right)\mathrm{Pr}({M}_{k})}{{\sum }_{{M}_{k}\in A}\mathrm{Pr}\left(D|{M}_{k}\right)\mathrm{Pr}({M}_{k})}.$$

This greatly reduces the number of models in the sum of Eq. (1) and now all that is required is a search strategy to identify models in set A. The other underlying principles are the two search strategy structures. The first principle of Occam's Window interpretation is related to the likelihood ratio of the posterior model $\mathrm{Pr}({M}_{0}|D)/\mathrm{Pr}({M}_{1}|D)$. Here, ${M}_{0}$ is a model with one less independent variable than ${M}_{1}$. The main idea shown in the figure below is that if there is evidence for ${M}_{0}$, then ${M}_{1}$ is rejected. But to reject ${M}_{0}$, we need strong evidence for the larger model, M_1_. If the evidence is not conclusive, none of the models can be rejected.

**Fig. 2:**
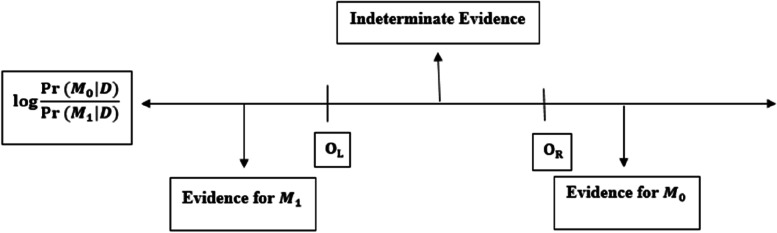
Occam’s Window and interpreting the Log Posterior Odds, $\mathrm{log}\left[pr({M}_{0}|D)/\mathrm{Pr}({M}_{1}|D)\right]$, where ${M}_{0}$ is a submodel of ${M}_{1}$

In Fig. 2, ${O}_{L}=-\mathrm{log}C$ *,* ${O}_{R}={O}_{L}^{-1}.$ The second principle is that if ${M}_{0}$ is rejected, then all the nested models in ${M}_{0}$, are rejected [10, 12, 18].

#### Markov Chain Monte Carlo Method (MCMC)

The MCMC method is one of the famous methods in probabilistic modeling, mainly the models whose parameter estimations do not have a closed form. This method estimates the parameters of interest based on random sampling. The MCMC algorithm is a combination of two separate methods, the Monte Carlo method and the Markov chain. In our problem, to use this method is to estimate the posterior probability of the model parameters in the Bayesian average, whose integration formula was shown in Eq. 1. There are many complex functions that cannot be sampled by conventional methods. The Monte Carlo sampling method is used to sample these complex functions. In fact, this method can provide a powerful tool that allows us to sample complex or high-dimensional functions, which tries to approximate the solution of the problem by generating random samples.

#### GBDT algorithm

GBDT is one of the new method of machine learning algorithm using multiple decision trees as the base learner. In GBDT, we combine weak learners so that they can reach to a level of strength. All trees are connected in series and each tree tries to minimize the error of the previous one [19]**.** In the GBDT, the number of base learners was 10,000 with the learning rate of 0.01. In our analysis, the minimum number of samples for leaf nodes was 10, the maximum depth of the tree was 4, and the node division impurity was 0.

#### Evaluation of the performance of fitted models with testing and training datasets

We used different criteria to evaluate the performance of models, including the average percentage of errors, MSE, average errors of different models, and graphical presentation in the calibration of the models. To calculate the average errors, first the error value, which is equal to the absolute value of the difference between the observed value and the estimated value was calculated. Then their average was calculated. Finally, the mean square errors was estimated as $MSE=\frac{\sum {\left(y-\widehat{y}\right)}^{2}}{N}$ for each model. One can use these three indicators to gauge the model fits. We also used the graphical calibration criterion, a coordination device, the x-axis of which is the observed Y and the y-axis is the estimated Y. The closer the line graph to the bisector of the first quadrant, the better the model fitting performance is.

## Results

### Descriptive statistics and bivariate analysis

Out of 4996 patients, 409(9%) died and 4587(91%) were discharged from the hospital. The mean and standard deviation of the discharged patients’ hospital stay compared to those who died is 6.36 ± 4.94 and 11.02 ± 10.19, respectively. Moreover, 2732 (55%) of participants were women and 2264 (45%) were men. The mean and standard deviation of the age were 55.3 ± 16.4 years for females and 58.8 ± 17.1 years for males. The mean (SD) of LOHS of the patients was 6.7 (5.7) days (6.51 days for women and 7.01 days for men). The longest LOHS for all patients was 85 days (68 days for women and 85 days for men). Of the total number of patients admitted to the hospital, 1109 (22.2%) had HTN, 1107 (22.2%) had diabetes, 663 (13.3%) had other chronic disorders, 91 (1.8%) had asthma, 49 (1%) had chronic nervous disorders, 34(0.7%) had COPD and 21(0.4%) had immunodeficiency. The Chi-square test was used to determine the relationship between the underlying disease and the dichotomized LOHS. Among the underlying diseases, diabetes (*p* = 0.001), chronic nervous disorders (*p* = 0.032), other COPD (*p* = 0.015) and HTN (*p* = 0.001) and other chronic disorders (*p* = 0.024) have had a significant association with dichotomized LOHS (Table 1).

**Table 1:** Demographic and comorbid of study subjects according to length of hospitalization (LOHS)

**Characteristic**	**LOHS(day)**		*X* ^2^	*p*- value
	** > 5**	** ≤ 5**		
**Sex**				
Male	1152(42.2)	1580 (57.8)		
Female	1049(46.3)	1215(53.7)	8.7	0.003
**Age group**				
≤50	646(34.8)	1209(65.2)		
51–64	683(46.8)	775(53.2)		
≥65	872(51.8)	811(48.2)	109.8	0.001
**Diabetes**				
No	1599(41.1)	2290(58.9)		
Yes	602(54.4)	505(45.6)	61.5	0.001
**Immunodeficiency**				
No	2188(44)	2787(56)		
Yes	13 (61.9)	8(38.1)	2.7	0.099
**Asthma**				
No	2155(43.9)	2750(56.1)		
Yes	46(50.5)	45(49.5)	1.5	0.208
**COPD**				
No	2179(43.9)	2783(56.1)		
Yes	22 (64.7)	12(35.3)	5.9	0.015
**Chronic Nervous Disorders**				
No	2172(43.9)	2775(56.1)		
Yes	29(59.2)	20(40.8)	4.5	0.032
**HTN**				
No	1612(41.5)	2275(58.5)		
Yes	589(53.1)	520(46.9)	47.4	0.001
**Other Chronic Disorders**				
No	1882(43.4)	2451(56.6)		
Yes	319(48.1)	344 (51.9)	5.1	0.024

Table 2 shows the mean of biological markers in relation to length of hospitalization (< = 5 days vs > 5 days). The mean of all biological markers except the ALT in patients who were hospitalized for more than five days is significantly higher than who were hospitalized for less than or equal to five days (*p* = 0.001).

**Table 2:** The Mean $\pm$ SD of biomarkers according to length of hospitalization

‌Biomarkers	LOHS(day)		Mean Difference (CI 95%)	*p*-value
	> 5	≤ 5		
	Mean ± SD	Mean ± SD		
PO2(mg)	91.4 ± 6.1	93.4 ± 4.3	2(1.7,2.2)	0.001
WBC(K/$\mu$ L)	7779.1 ± 15791.3	6537.2 ± 4504.1	-1241.9 (-1856.8,-626.9)	0.001
ALT(IU/L)	40.1 ± 52.8	39.8 ± 74.8	-0.3 (-3.9,3.3)	0.870
AST(U/L)	52.6 ± 40.4	46.3 ± 35.1	-6.3 (-8.3,-4.2)	0.001
ESR(mm/hr)	43.4 ± 28.7	36.4 ± 26.1	-7 (-8.5,-5.4)	0.001
CRP(mg/L)	72.9 ± 60.7	51.7 ± 49.1	-21.2 (-24.2,-18.1)	0.001
BUN(mg/dl)	23.1 ± 15.6	19.1 ± 12.1	-4 (-4.7,-3.2)	0.001
ALP(IU/L)	202.3 ± 155.2	189.7 ± 112.2	-12.6 (-20.1,-5.1)	0.001
NLR	5.4 ± 4.3	4.3 ± 3.2	-1.1(-1.3,-0.8)	0.001

### Findings of fitting classical regression models

To develop the models, at this stage, the models were fitted with the training data sets and evaluated with the test data sets. The regression coefficients based on three methods of stepwise, AIC and BIC of the fitted classical models were shown in Table 3.

**Table 3:** The regression coefficients of the classic linear regression model of prognostic variables in predicting the length of hospital stay (LOHS) of covid-19 patients

Independent	Stepwise			AIC			BIC		
Variables	B	S.E	*p*-value	B	S.E	*p*-value	B	S.E	*p*-value
Constant	10.33	1.10	0.001	3.008	0.232	0.001	2.97	0.22	0.001
NLR	0.056	0.016	0.001	0.059	0.016	0.001	0.053	0.016	0.001
Anorexia(yes vs no)	-0.411	0.155	0.008	-0.42	0.15	0.007	${-}^{+}$	${-}^{+}$	${-}^{+}$
BUN(mg/dl)	0.013	0.0046	0.003	0.013	0.0046	0.004	0.013	0.0045	0.004
CRP(mg/L)	0.0068	0.001	0.001	0.008	0.001	0.001	0.0078	0.001	0.001
Respiratory distress(yes vs no)	0.510	0.1171	0.001	0.58	0.1174	0.001	0.615	0.117	0.001
AST(IU/l)	0.0055	0.0015	0.001	0.0092	0.0024	0.001	0.0051	0.0015	0.001
Diabetes(yes vs no)	0.462	0.1424	0.001	${-}^{+}$	${-}^{+}$	${-}^{+}$	${-}^{+}$	${-}^{+}$	${-}^{+}$
WBC(k/$\mu l)$	0.000023	0.000004	0.001	0.00002	0.000004	0.001	0.00002	0.000004	0.001
Discharge status (death vs alive)	-0.82	0.2414	0.001	-0.67	0.24	0.005	${-}^{+}$	${-}^{+}$	${-}^{+}$
ICU(yes vs no)	4.6	0.2737	0.001	4.83	0.27	0.001	4.63	0.253	0.001
Age(year)	0.023	0.0036	0.001	0.023	0.0038	0.001	0.025	0.0036	0.001
Chronic Nervous Disorders(yes vs no)	1.45	0.631	0.021	1.51	0.636	0.017	${-}^{+}$	${-}^{+}$	${-}^{+}$
PO2(mmHg)	-0.077	0.0112	0.001	${-}^{+}$	${-}^{+}$	${-}^{+}$	${-}^{+}$	${-}^{+}$	${-}^{+}$
Chest Pain(yes vs no)	1.07	0.367	0.003	1.046	0.36	0.005	${-}^{+}$	${-}^{+}$	${-}^{+}$
HTN(yes vs no)	${-}^{+}$	${-}^{+}$	${-}^{+}$	0.26	0.145	0.065	${-}^{+}$	${-}^{+}$	${-}^{+}$
ALT(IU/l)	${-}^{+}$	${-}^{+}$	${-}^{+}$	-0.0044	0.0023	0.058	${-}^{+}$	${-}^{+}$	${-}^{+}$
Consciousness(yes vs no)	${-}^{+}$	${-}^{+}$	${-}^{+}$	0.73	0.41	0.077	${-}^{+}$	${-}^{+}$	${-}^{+}$

${-}^{+}$ The variable is not available in the model

In the stepwise model, the ${R}^{2}$ value is 0.168 and the adjusted ${R}^{2}$ value was 0.165. As shown in Table 3, the biomarkers NLR, AST, WBC, CRP, and BUN, among the clinical symptoms of anorexia, respiratory distress, and chest pain and the underlying diabetes, chronic nervous disorders, and also variables such as age, hospitalization in ICU, and PO2, had a positive significant relationship with LOHS. To fit the AIC model, the variables were entered into the model one by one, and then the AIC value of the model was calculated. Finally, the model with the lowest AIC value was selected as the best model, ${R}^{2}$= 0.168 and adjusted ${R}^{2}$ is equal to 0.165.

In this model, clinical symptoms such as chest pain, respiratory distress, level of consciousness, and anorexia, biomarkers such as CRP, WBC, NLR, AST, BUN, and ALT, underlying diseases, chronic nervous disorders and HTN, as well as variables such as age, hospitalization in ICU, and discharge status a statistically significant correlation with LOHS. To fit the BIC model, the variables were entered into the model one by one, and then the BIC value of the model was calculated. Finally, the model with the lowest BIC value was selected as the best model. The ${R}^{2}$ value is 0.160 and the adjusted ${R}^{2}$ value is 0.158. In this model, biomarkers such as CRP, AST, WBC, BUN, and NLR, clinical symptoms of respiratory distress, and demographic characteristics such as age, as well as the binary variable of hospitalization in ICU, were significantly predictive of LOHS. A similar and consistency was observed in the coefficients of the models with the three classical methods. The adjusted ${R}^{2}$ in the model of the stepwise method and AIC is slightly higher than that of the BIC method.

### Findings of Bayesian model averaging

#### Fitting the Occam's Window model

To fit BMA, among all the models, 69 models with higher prediction probability have been selected. In Table 4, models 1 to 5 were obtained from the combination of 69 models. Among these models, the 5 models were selected with the high posterior probability with the highest one was model 1.

**Table 4:** Coefficients of the Bayesian linear regression model using Occam's Window method in predicting the length of hospital stay of covid-19 patients

Independent variables	*P* ≠ 0	Model1	Model2	Model3	Model4	Model5
Constant	100	10.31	10.36	10.14	10.19	10.3
Age(year)	100	0.022	0.022	0.024	0.024	0.026
Sex(male vs female)	0	0	0	0	0	0
ICU(yes vs no)	100	4.6	4.5	4.3	4.3	4.6
Fever(yes vs no)	0	0	0	0	0	0
Cough(yes vs no)	0	0	0	0	0	0
Musculoskeletal pain(yes vs no)	0	0	0	0	0	0
Respiratory(yes vs no)	100	0.52	0.5	0.52	0.5	0.52
Consciousness(yes vs no)	1.1	0	0	0	0	0
Smell(yes vs no)	0	0	0	0	0	0
Taste(yes vs no)	0	0	0	0	0	0
Seizure(yes vs no)	0	0	0	0	0	0
Abdominal pain(yes vs no)	0	0	0	0	0	0
Nausea(yes vs no)	0	0	0	0	0	0
Vomiting(yes vs no)	0	0	0	0	0	0
Anorexia(yes vs no)	23.2	0	0	0	0	0
Headache(yes vs no)	0	0	0	0	0	0
Dizziness(yes vs no)	0	0	0	0	0	0
Chest pain(yes vs no)	45.7	0	1.028	0	1	0
PO2(mmHg)	100	-0.077	-0.078	-0.074	-0.074	-0.077
Diabetes(yes vs no)	83	0.48	0.45	0.5	0.47	0.51
Immunodeficiency(yes vs no)	0	0	0	0	0	0
Asthma(yes vs no)	0	0	0	0	0	0
Chronic nervous disorders(yes vs no)	9.2	0	0	0	0	0
Other chronic disorders(yes vs no)	0	0	0	0	0	0
HTN(yes vs no)	0	0	0	0	0	0
Discharge status(yes vs no)	53.1	-0.788	-0.804	0	0	-0.62
WBC(k/*µ*l)	100	0.00002	0.00002	0.00002	0.00002	0.00002
ALT(IU/l)	0	0	0	0	0	0
AST(U/l)	85.2	0.005	0.005	0.005	0.005	0.005
ESR(mm/hr)	0	0	0	0	0	0
CRP(mg/L)	100	0.0067	0.0068	0.0067	0.0068	0.0069
BUN(mg/dl)	57	0.014	0.014	0	0	0
ALP(U/L)	0	0	0	0	0	0
COPD(yes vs no)	0	0	0	0	0	0
NLR	85	0.0544	0.054	0.053	0.053	0.057
n Var		11	12	9	10	10
*R* ^2^		0.174	0.176	0.171	0.172	0.172
BIC		-673.4	-673	-672.8	-672	-671.6
Post prob		0.087	0.07	0.063	0.042	0.034

#### Fitting the BMA using Monte Carlo Markov chain (MCMC)

To fit the model, MCMC simulation only specifies the selection of predictor variables but does not specify the regression coefficients of the explanatory variables. Among all the models, 336 models have been placed in the chain, and 5 models have been obtained by combining these models. The models, the posterior probability of each predictor variable, as well as the posterior probability of the models are presented in Table 5. Among the 5 models obtained, model 1 was selected since it has a higher posterior probability.

**Table 5:** Coefficients of the Bayesian linear regression model using the MCMC method of variables predicting the length of hospital stay of Covid-19 patients

Independent variables	*P* ≠ 0	Model1	Model2	Model3	Model4	Model5
Age(year)	100	X	X	X	X	X
Sex(male vs female)	0.86	0	0	0	0	0
ICU(yes vs no)	100	X	X	X	X	X
Fever(yes vs no)	0	0	0	0	0	0
Cough(yes vs no)	0	0	0	0	0	0
Musculoskeletal pain(yes vs no)	0	0	0	0	0	0
Respiratory(yes vs no)	100	X	X	X	X	X
Consciousness(yes vs no)	4.7	0	0	0	0	0
Smell(yes vs no)	0	0	0	0	0	0
Taste(yes vs no)	0	0	0	0	0	0
Seizure(yes vs no)	0	0	0	0	0	0
Abdominal pain(yes vs no)	0	0	0	0	0	0
Nausea(yes vs no)	0	0	0	0	0	0
Vomiting(yes vs no)	0	0	0	0	0	0
Anorexia(yes vs no)	15	0	0	0	0	0
Headache(yes vs no)	0	0	0	0	0	0
Dizziness(yes vs no)	0	0	0	0	0	0
Chest pain(yes vs no)	45	0	0	X	0	X
PO2(mmHg)	100	X	X	X	X	X
Diabetes(yes vs no)	86	X	X	0	0	X
Immunodeficiency(yes vs no)	0	0	0	0	0	0
Asthma(yes vs no)	0	0	0	0	0	0
Chronic nervous disorders(yes vs no)	48	0	X	0	X	X
Other chronic disorders(yes vs no)	0	0	0	0	0	0
HTN(yes vs no)	2.3	0	0	0	0	0
Discharge status(death vs alive)	76	X	X	0	0	0
WBC(k/$\mu l)$	100	X	X	X	X	X
ALT(IU/l)	14.1	0	0	0	0	0
AST(IU/l)	100	X	X	X	X	X
ESR(mm/hr)	0	0	0	0	0	0
CRP(mg/L)	100	X	X	X	X	X
BUN(mg/dl)	100	X	X	X	X	X
ALP(U/L)	0	0	0	0	0	0
COPD(yes vs no)	0	0	0	0	0	0
NLR	100	X	X	X	X	X
nVar		11	12	10	10	12
Post prob		0.34	0.2028	0.074	0.0638	0.042

### Model fit based on GBDT

**Fig. 3:**
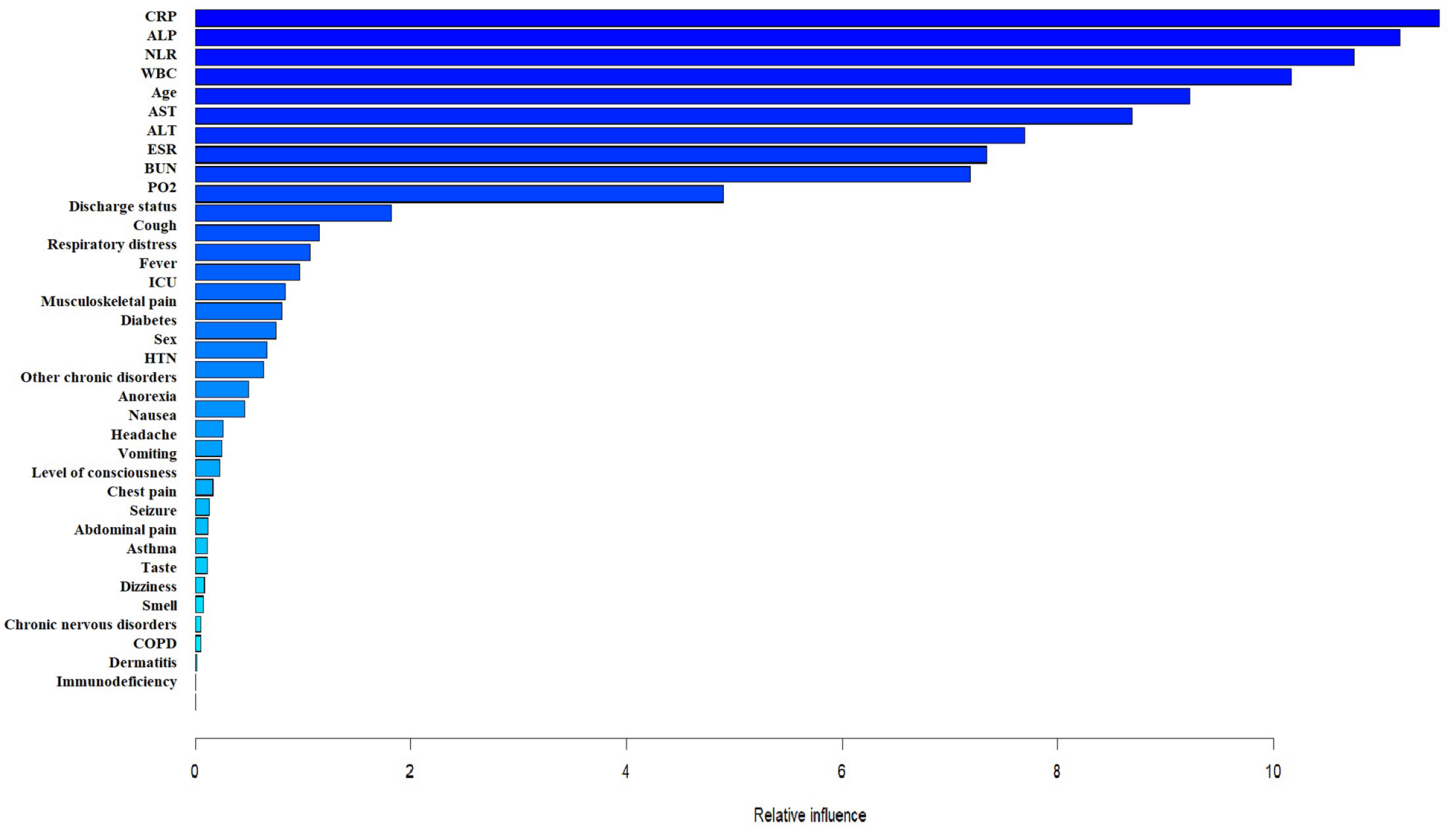
The relative effectiveness of the variables on the LOHS in GBDT model

Figure 3 shows the influence of variables on LOHS. As one can see CRP, ALP, NLR, WBC, age, AST, ALT, ESR, BUN, and PO2 variables have had the greatest effect. In addition, in GBDT algorithm, the value of R^2^ = 0.64 that was relatively higher than the classical regression model and BAM.

**Fig. 4:**
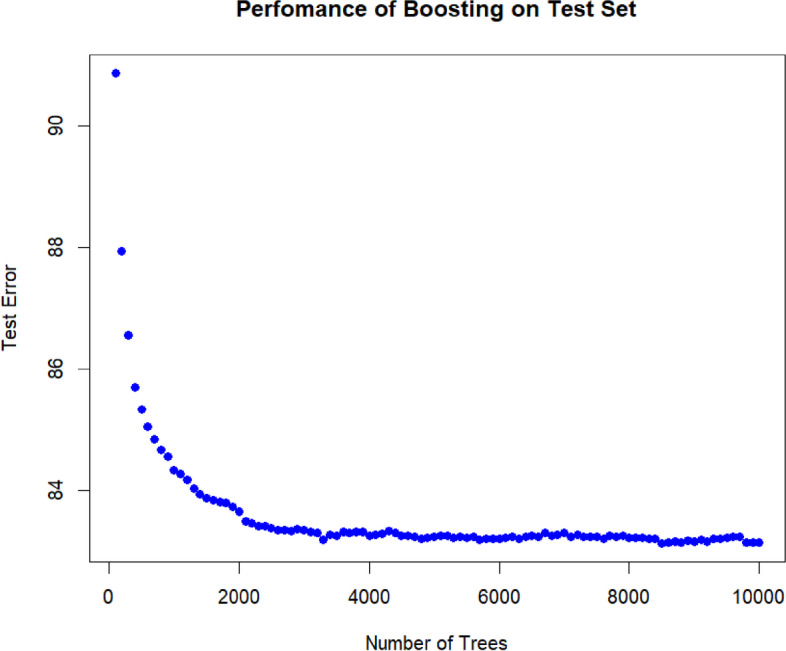
Performance of boosting on test set

As one can see in Fig. 4, the MSE value of the model has decreased while the tree has had an upward movement. Obviously, this rise started from almost 5000 trees to 10,000 trees, with nearly equal MSE values.

### Comparison of predictive performance of fitted models in testing and training datasets

The performance of the fitted model predictors was investigated in 80% of training data and 20% of the remaining testing data and the efficiency of models were evaluated using the fitting indicators, such as a graphic criterion, the calibration chart, the average percentage of errors, the average errors, and also the magnitude of $MSE=\frac{\sum {\left(y-\widehat{y}\right)}^{2}}{N}$ for each of the classical and BMA models.

Table 6 shows the average percentage errors, MSE and the average errors of different models both in the training and the testing datasets. In the testing dataset, the average percentage error of the fitted Occam's Window model is 60.53%, which is lower than all other models, and then the MCMC is 61.89%. Furthermore, Overall, in the testing data, the MSE of all fitted model were relatively high. The MSE value of Occam's Window model is the lowest, and the stepwise model is the highest. The average error of Occam's Window is the lowest and the stepwise method is the highest. In the testing data, the mean percentage errors, the mean of errors and the MSE in the GBDT method are almost similar to the classical regression methods. However, in the training data, as one expects, the MSE and the mean of errors was substantially decreased for all fitted models. The lowest MSE (5.5) and mean of error (1.73) belongs to the GBDT. The BMA performance was better than classical regression model but not GBDT in the training dataset.

**Table 6:** Mean percent of errors, MSE, and mean of errors in classical models, Bayesian averaging model and GBDT algorithm according to the training and the testing datasets

	Training			Training		
Fitted models	Mean percentage errors	MSE	Mean of errors	Mean percentage errors	MSE	Mean of errors
Stepwise	61.33	12.90	2.618	89.40	92.50	4.92
AIC	63.40	13.30	2.691	86.29	85.83	4.58
BIC	61.77	13.20	2.633	87.60	85.68	4.62
Occam’s Window	54.40	10.97	2.003	60.53	75.82	3.57
MCMC	55.40	10.95	2.008	61.89	78.67	3.57
GBDT	40.60	5.50	1.730	87.08	83.15	4.61

**Fig. 5:**
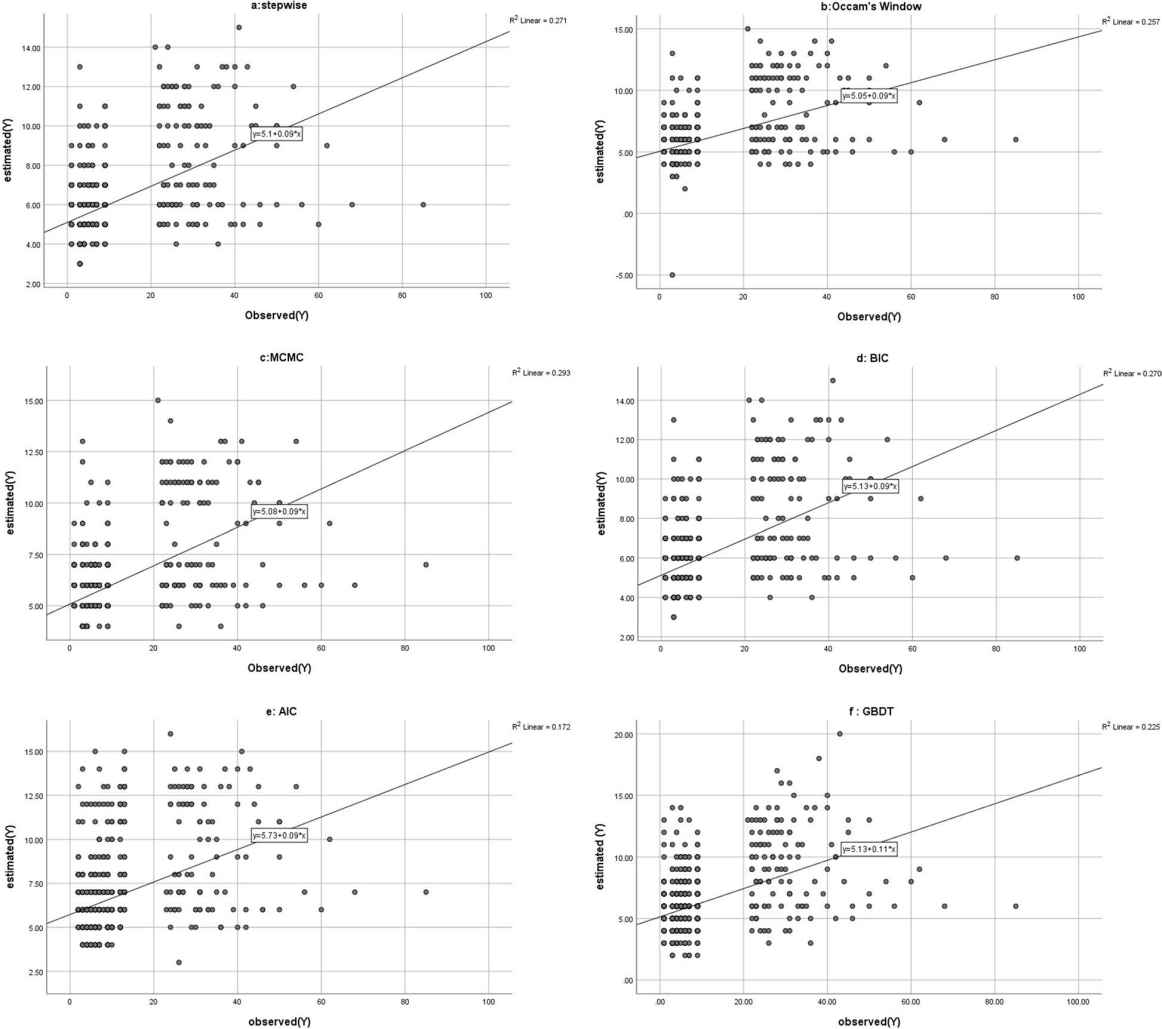
The calibration diagram of the six different models in testing dataset in six panels (**a**, **b**, **c**, **d**, **e**, **f**)

According to the fitting indicators in Fig. 5 for 20% of the testing data, the fitted model of Occam's Window had a better fit than other models. To evaluate the predictive power of the models using testing data, a graphical criterion of the predictive performance was used to determine whether the predictions were well calibrated. In this graph, the x-axis is the observed value of y and the y-axis is the estimated value of y. In the calibration plan, the full calibration is the 45-degree line, and therefore, the closer the model calibration line is to the 45-degree line, the better the calibration is. The calibration diagram for different models was shown in 6 panels of Fig. 5. In comparing the efficiency of the models, Occam's Window BMA has a higher predictive power than other methods. As one can see, the slope of the line of all the models was rather similar and the width from the origin of Occam's Window model is less than all the models. The predictive power of GBDT model in testing dataset is almost similar to AIC and BIC methods.

## Discussion

In this study, the factors affecting the LOHS of COVID-19 were identified through statistical modeling using the classical method, the BMA and GBDT algorithm. The risk factors such as hospitalization in the ICU, age, diabetes, and biomarkers such as CRP, PO2, WBC, NLR, AST, BUN, and respiratory distress had a significant relationship with LOHS. The length of actual hospitalization and its affecting factors are of particular importance for managers and health policymakers for better allocation of health resources due to limited financial resources. Several studies that predict the affecting factors of the length of hospitalization of COVID-19 mainly used the classical statistical method, which is in agreement with the results of our study to large extent [5, 20, 21].

Based on our findings, hospitalization in the intensive care unit (ICU) is one of the main factor during the patients’ referral to hospitals. This variable has been included in the 6 proposed models as one of the main predictor in the current study. Out of all the patients with COVID-19, 293 (6%) people were hospitalized in ICU. The average duration of patients’ hospital stays in ICU was significantly higher than in normal hospital wards. In many other studies, such as Zhang, which is a systematic review and meta-analysis of 45 studies including 4203 patients, hospitalization in the ICU has been among the influencing factor on LOHS [22]. In another study by Al-Harthi in Saudi Arabia, out of 352 malignant patients with COVID-19, hospitalization in ICU is one of the main factors in the mortality of patients [23].

A multicenter prospective study was also conducted on children with SARS-COV-2 infection in 52 hospitals in Spain. Age, neutrophilia, and PO2 were significant predictors on the LOHS [24]. These factors in the present study were the main factors in our six developed models. In a study conducted by Hadley et al. (2022) in the United States, age plays a crucial role in the LOHS [25], which was also one of the main independent variables in all 6 of our proposed models. The mechanism of renal dysfunction caused by SARS-CoV-2 is still unknown. More and more reports have shown that SARS-CoV-2 plays a pathogenic role in COVID-19 patients through binding to the angiotensin-converting enzyme (ACE) 2 receptor [26].

In current study, the effect of the ratio of neutrophils to lymphocytes (NLR) was present and significant in our six developed models as well. In a cross-sectional study conducted by Birhanu et al. (2019) in Ethiopia, in the analysis of a total of 394 hospitalized for the coronavirus disease, it was reported that NLR played an important role in predicting hospitalization of COVID-19 [5]. So after the start of the COVID-19 pandemic, it was observed that NLR was much higher in severe or critically ill patients compared to outpatients. It has been shown that NLR is a reliable indicator to determine the severity of the disease in COVID-19 [27] that is also consistent with our findings. Many mechanisms have been hypothesized regarding the response of neutrophils and lymphocytes to coronavirus infection. Neutrophils activate the immune system and release reactive oxygen species that can damage cellular DNA and release the virus from cells, which are then targeted by antibodies. In addition, neutrophils produce various cytokines and effector molecules. On the other hand, although viral infection itself primarily stimulates a lymphocytic response, systemic inflammation, particularly interleukin-6, paradoxically reduces lymphocyte numbers and the resulting cellular immunity. Both of these factors lead to an increase in NLR. Therefore, higher NLR predicts the severity of inflammation [28, 29]. In other cross-sectional study conducted in Ethiopia, the role of NLR as a predictor of the severity and mortality of COVID-19 patients has been reported [30]. In our findings, NLR is one of the main factors in predicting the LOHS with its effects being significant in our six developed models.

Clinical studies showed that the fluctuation of some blood markers may be related to the degree of severity and mortality COVID-19 among patients. Among these clinical parameters, serum C-reactive protein (CRP) has been found as an important marker that changes significantly in severe cases [31]. CRP is a protein produced by the liver that acts as an early indicator of infection and inflammation [32] in blood. The normal concentration of CRP is less than 10 mg/L. However, it increases rapidly within 6 to 8 h and peaks at 48 h after illness onset [33]. It is alive for about 19 h, and its concentration decreases with the end of the inflammatory stages and the recovery of the patient. CRP preferentially binds to phosphocholine, which is highly expressed on the surface of damaged cells [34]. This binding activates the classical complement pathway of the immune system and modulates phagocytic activity to clear microbes and damaged cells from the organism. Once inflammation or tissue damage has resolved, CRP concentrations decrease, making it a useful marker for monitoring disease severity [33]. In a retrospective study on 429 patients with COVID-19 by Mahmoud Sadeghi et al. in Iran in 2020, CRP could be used as an independent factor in predicting the severity of COVID-19. Also, patients with CRP < 64.75 mg/L were more likely to have severe complications. As a result, serum levels of CRP can predict the severity and progression of the disease in patients with COVID-19 [35]. In another retrospective study in the United States, CRP was observed as one of the most important predictors of LOHS [36]. This is also in concordance with our findings.

Furthermore, in a review study, a total of 14 studies reported the results of 4659 COVID-19 patients, diabetes was one of the main predicting variables in LOHS [7]. This variable also was shown as one of the significant determinants in predicting the LOHS in our four built models. The presence of risk factors investigated as predictors of LOHS in the present study may be considered an indicator of the severity of the disease, which led to a longer period of hospitalization for COVID-19. That is, the average duration of LOHS for diabetic patients was longer than that of patients without diabetes. which is consistent with al-Salamah's [37] study in France.

In the current study, the average LOHS of patients with COVID-19 was approximately 7 days, which is in agreement with the results of other studies [21, 38–41]. In a study conducted in Brazil, the average LOHS was 8.6 days [38]. It is consistent with the studies of Abdullah Al-Ahmari et al. [39], Thiruvengadam et al. [21], each with a length of hospitalization of 7 days. The other systematic review was conducted in China, out of 52 studies, composed of 46 studies inside China and 6 studies outside of China. The median of LOHS was between 4 and 21 days outside China, and between 4 and 53 days inside China [41]. While in our study the LOHS varied from 1 to 85 days and the median was 5. The difference may be explained by treatment protocol, the difference in the severity of the disease, and the difference in the level of immunity and genetic factors in China and Iran. In contrast, in another study conducted in China, the median LOHS was reported to be about 13 days [40], which was higher than the median hospitalization in our study.

The findings of this study have shown that the Bayesian averaging approach can be successfully used as an alternative classical regression method in the development of prognostic models for the length of hospitalization of COVID-19. The results of the present study show that, despite having a higher number of explanatory variables, classical models have lower ${R}^{2}$ and adjusted ${R}^{2}$ than the BMA. In the present study, when we look at the six proposed models, the explanatory significant variables of the models were very similar. With a closer look, one can see that there are many differences between the models, such as Occam's Window model and the Monte Carlo Markov chain model of the underlying disease comorbidity. Diabetes plays an important role in the LOHS, while in the forward models based on the AIC, BIC approach, it was not significant. Also, the underlying chronic neurological disorders, and clinical symptoms such as anorexia, were significant in stepwise and forward models based on the AIC approach, but do not have a significant effect in our proposed models in Bayesian averaging method.

Also, the variables of ALP, ALT and ESR in the GBDT model show us that these variables have a significant impact on determining the length of hospitalization, this variable was not significant at all in the other proposed models. Based on the findings of the present study, although the GBDT method has a higher ${R}^{2}$ than other fitted models, its predictive power, average percentage of errors, and MSE are almost similar to traditional regression models. The reason for the higher R^2^ value in GBDT compared to regression with the same MSE is due to the nature of the algorithms themselves. Regression is a linear algorithm and attempts to find the optimal line of best fit through the data points. This can result in a lower R-square value because it only considers the linear relationship between the independent and dependent variables. On the other hand, GBDT is a non-linear algorithm that can capture more complex relationships between variables. It can build a tree structure through iterative learning and reinforcement, which helps capture more nuisances in the data that linear regression might miss. Therefore, the *R*^2^ value in GBDT is higher than traditional regression due to the ability of the algorithm to model nonlinear relationships. However, since MSE measures how far the predicted values are from the actual values, and does not measure the nature of the recorded relationship between the variables, it can be the same for both regression and GBDT [42]. At the same time, other studies comparing other machine learning (ML) algorithms have found that the GBDT method has a higher efficiency and a lower error percentage compared to other ML algorithms [43]. In our results, there are many similarities in the selected variables in different models to some extent. The observed differences among different models can be considered as the result of the difference in the mechanism of the selection methods of variables in different models. The predictive performance in terms of MSE is relative high for all fitted models in the testing dataset. This perhaps is due to relative large unexplained variability of LOHS that was not captured by our proposed models. This unexplained variability in part may depend on subjective view of doctors and patients’ own view in LOHS that we were not able to measure them or any other unmeasured indigenous variables that were not included into the fitted models. However, in comparison with the fitted performance in training dataset, the MSE and the mean of errors were substantially decreased and the lowest MSE belong to GBDT. This indicates that the GBDT algorithm captures more non-linear pattern of all explanatory variables than traditional regression model and BMA in training dataset but not in testing dataset.

In our study, the six proposed models were drawn to evaluate the calibration chart using testing data. A linear graph whose slope is one and the intercept from the origin is zero is a complete calibration. Among the six panels of these graphs shown, Bayesian averaging has been able to have a better calibration than the calibration graphs of classical models and GBDT. These findings are consistent with the study of Raftery et al. [12], which indicated the performance BMA is better than classical models. Based on the findings of the present study, the BMA based on Occam's Window has a lower percentage of error and MSE than other models. However, so far there was no data to compare the BMA with new method of GBDT algorithm in the published studies.

The present study is superior to the previous studies that assessed the LOHS from several points of view. First, a large database was used that definitely reduces the sampling variation. Second, in our study, the posterior probability of the main predictors of LOHS was calculated directly using the Bayesian method, while the classical method is not able to calculate this probability. Third, in this study, the BMA estimates the coefficients of predictive factors with a higher certainty than the classical regression models. Fourth, we implemented the BMA in the training data. In addition, the predictive performance of the developed models was measured using the testing dataset in terms of calibration and estimation of errors while in many studies in this medical context, the model fitting criteria have not been reported in their findings not only in testing data but also in training data. Another innovation aspect of the study was the comparing BMA with the new method of GBDT and traditional regression approach in predictors of LOHS of COVID-19 and our results explored that the performance of BMA outreaches to other models.

In the present study, a novel forecasting model with the BMA method was developed to predict the LOHS of COVID -19. The findings of the present study from the statistical model of BMA and the new method of GBDT algorithm of machine learning regarding the LOHS and its influencing factors are a new innovation. Despite the fact that many studies have been conducted on the LOHS of COVID-19 in the world, this type of modeling based on BMA was not used. Based on the findings of this study, we expect that by controlling these factors the LOHS will be reduced and the burden of expenses caused by this common disease on the family and the health services system will be minimized.

## Limitations

The BMA is designed so that R is the only mainstream statistical platform that provides a set of methods for performing BMA analysis [44]. We could not collect powerful data from multiple centers to train the model. Thus, we should be cautious in generalizing the results. However, our database was from a referral center hospital that covers over half a million of the general population in the north of Iran. Moreover, because the database was collected retrospectively, some data had missing values. However, we used the advanced statistical method of multiple imputations to impute the missing value. In addition, one might argue that the LOHS is a subjective indicator and may have a slightly different understanding of different doctors' and patients' beliefs own state. It is affected by a variety of subjective matters, the availability of hospital resources, treatment methods, and patients’ views. However, the subjective errors of observed LOHS are non-differential to the level of biomarkers on the first day of hospitalization. Thus, it does not create an association. However, it may dilute the real association and the effect size of interest and lead to the performance of models being diminished. Finally, we observed a large variability of LOHS, and the major part of this variability cannot be explained by the explanatory variables in the models that we have built. Perhaps a part of the unexplained variability of LOHS might be due to subjective indicators of LOHS and other indigenous variables that were not measured in the current study. This related issue caused a relatively high MSE in the evaluation of the performance of models in the testing dataset.

## Conclusion

This study showed that BMA can integrate superior model that can have a higher certainty than traditional regression models in detecting the predictors of LOHS. We found the relative large unexplained variability of LOHS for all fitted model. Our findings indicated that the BMA using Occam's Window method has higher superiority in explaining the length of hospitalization for COVID-19 than GBDT algorithm and other traditional regression models in the final evaluation of predictive performance in testing dataset. While GBDT has better predictive performance in the training dataset than other models. The clinical results of this study showed that the hospitalization in ICU, age, diabetes, PO2, WBC, BUN AST, CRP, and NLR are the most influencing factors on the LOHS with COVID-19. Thus, by controlling these factors, it may be possible to reduce the length of hospitalization due to COVID -19.

## Data Availability

The data underlying this manuscript are available from corresponding author upon the reason able request.
